# Improved Outcomes of Combined Main Branch Stenting and Side Branch Drug-Coated Balloon versus Two-Stent Strategy in Patients with Left Main Bifurcation Lesions

**DOI:** 10.1155/2022/8250057

**Published:** 2022-01-11

**Authors:** Hengdao Liu, Hailong Tao, Xufei Han, Yang Lu, Xiaofei Xue, Ruihan Feng, Fenghua Lv, Yanwei Liu, Hongrui Jin, Lianjie Li, Heping Gu

**Affiliations:** ^1^Department of Cardiology, The First Affiliated Hospital of Zhengzhou University, Zhengzhou 450052, Henan, China; ^2^Department of Cardiology, The First Affiliated Hospital of Xinxiang Medical University, Xinxiang 453100, Henan, China; ^3^Department of Infectious Disease, The First Affiliated Hospital of Xinxiang Medical University, Xinxiang, Henan 453100, China; ^4^Department of Magnetic Resonance, The First Affiliated Hospital of Zhengzhou University, Zhengzhou 450052, China; ^5^Department of Cardiology, Xichuan Second People's Hospital, Nanyang 474450, Henan, China

## Abstract

**Background:**

Drug-eluting stent (DES) plus drug-coated balloon (DCB) is a safe and effective treatment strategy for coronary artery bifurcation lesions, but there is no report about this strategy being used for left main (LM) bifurcation lesions. We aim to explore the efficacy and safety of DES plus DCB in the treatment of LM bifurcation lesions.

**Methods:**

A total of 100 patients diagnosed with LM bifurcation lesions by coronary angiography were retrospectively enrolled at our center from January 2018 to December 2019. They received either a two-stent strategy or a main branch (MB) stenting plus side branch (SB) DCB strategy and were accordingly divided into the 2-DES group and the DES + DCB group. Patients treated with DES + DCB were compared with a cohort of matched patients treated with a 2-DES strategy. Clinical data was collected and quantitative coronary analysis was performed.

**Results:**

For immediate postoperative angiography, though the two groups had no differences in the minimal luminal diameter (MLD) and luminal stenosis of MB, the DES + DCB group had significantly lower SB ostial MLD and a higher degree of residual lumen stenosis than the 2-DES group (*P* < 0.05). At the time of follow-up, the SB ostial MLD of the DES + DCB group was higher than that of the 2-DES group, but lumen stenosis, late lumen loss (LLL), and LLL at the distal end of the left MB were all smaller than those of the 2-DES group (*Ps* < 0.05). Furthermore, the incidence of lumen restenosis and MACE between the two groups had no significance.

**Conclusion:**

The combination of DES and DCB is relatively safe and effective for the treatment of LM bifurcation lesions, and this strategy seems to have advantages in reducing LLL at the SB ostium.

## 1. Introduction

The left main coronary artery (LMCA) affects at least 75% of the blood supply to the left ventricular myocardium. Therefore, lesions in the left main stem often indicate a higher risk of poor prognosis [1, 2]. Among all the lesion types of the LMCA, the LM bifurcation lesion is the most common type, accounting for about 50% [1]. Percutaneous coronary intervention (PCI) is one of the most commonly used approaches for the treatment of LM bifurcation lesions. Studies have shown that coronary artery bypass grafting (CABG) and PCI with a new generation of drug-eluting stents (DESs) have similar clinical prognosis for patients with low-risk and intermediate-risk LM bifurcation lesions (SYNTAX score ≤32 points) [3, 4]. Current PCI treatments for LM bifurcation lesions include single-stent implantation and double-stent implantation. The latest meta-analysis shows that compared to the double-stent strategy, the single-stent strategy has the advantages of a simpler operation and less target lesion revascularization (TLR) during follow-up [5]. Nevertheless, there are still some patients who need to implant a second stent in the side branch (SB) due to various reasons [6]. This approach is supported by the latest guidelines and expert consensus, which recommends SB stenting, when necessary, as the basic treatment strategy for most patients with bifurcation lesions [7, 8].

In recent years, the application of drug-coated balloons (DCBs) in coronary bifurcation lesions has attracted widespread attention, and the use of combined main branch (MB) DES implantation and SB DCB dilation as a new treatment strategy is expanding. A number of clinical studies have shown that DES plus DCB is a safe and effective strategy for the treatment of coronary artery bifurcation lesions because it can effectively reduce the use of additional stents in SB and has less late lumen loss (LLL) in SB during angiographic follow-up [9–11]. However, these studies included fewer lesions in the left main bifurcation and lacked a comparison with the traditional double-stent strategy. Thus, this study aimed to explore the effectiveness and safety of MB stenting plus SB DCB in the treatment of left main bifurcation lesions by comparing it with the traditional double-stent strategy and to provide a reference for clinical intervention of LM bifurcation lesions.

## 2. Materials and Methods

### 2.1. Study Population

A total of 100 patients were retrospectively enrolled from January 2018 to December 2019 in a single center (the First Affiliated Hospital of Zhengzhou University, Zhengzhou, China). They were diagnosed with true left main bifurcation lesions by coronary angiography (CAG). According to the interventional treatment plan, all patients received either a two-stent strategy (2-DES group) or a main branch (MB) stenting plus side branch (SB) DCB strategy (DES + DCB group). In this study, patients treated with DES + DCB were compared with a cohort of matched patients treated with the 2-DES strategy.

The inclusion criteria were as follows: (1) according to the definition of the Medina classification [12], patients were confirmed to have true left main bifurcation lesions by CAG, and (2) the left main stem had no unobstructed bridging vessels, and there was no good collateral circulation that shunts right to left. The exclusion criteria were as follows: (1) patients who had received CABG in the past, (2) patients who had a stent implantation in the left main bifurcation before, (3) with chronic total occlusion, (4) with severely calcified lesions that required rotational atherectomy, (5) with acute myocardial infarction (MI), and (6) with primary cardiomyopathy.

Before the operation, all patients and their families signed an informed consent form and an authorization letter for the interventional treatment of coronary artery disease.

### 2.2. Data Collection

Basic patient information was collected, including sex, age, clinical diagnosis, history of hypertension, history of diabetes, history of hyperlipidemia, history of MI, history of PCI, history of smoking, and left ventricular ejection fraction (LVEF) value.

### 2.3. PCI Procedure

All patients received dual antiplatelet therapy (DAPT) preoperatively (loading dose: aspirin 300 mg + clopidogrel 300 mg or aspirin 300 mg + ticagrelor 180 mg; maintenance dose: aspirin 100 mg/d + clopidogrel 75 mg/d or aspirin 100 mg/d + ticagrelor 90 mg, twice a day) and statin therapy preoperatively.

To clearly show the left main bifurcation lesion and other coronary artery lesions, multiposition imaging was performed using radial or femoral artery access. The blood perfusion of each diseased vessel was evaluated according to the thrombolysis in myocardial infarction (TIMI) flow grade. Surgeons selected surgical methods based on the characteristics of specific lesions and employed intracavitary imaging to assist decision-making when necessary. In interventional therapy, the left main stem and left anterior descending (LAD) artery were usually regarded as MB, and the left circumflex (LCX) artery was regarded as SB.

The PCI approach was as follows: for the DES + DCB group, first the lesion was fully predilated and DES was implanted in the MB, then the DES metal mesh was dilated at the bifurcation and the DCB was placed in the SB. Subsequently, the balloon was sent to the MB and SB for final kissing balloon inflation, and finally, the proximal optimization technology (POT) was performed by sending the postdilated balloon to the proximal end of the MB stent. If the angle between MB and SB was large (>90°), the SB DCB was positioned before the implantation of the MB stent because it was expected that it would be difficult to exchange the SB guidewire, or if it is difficult for the DCB to enter the SB after the MB stent is implanted. For the 2-DES group, after sufficient predilation of the target lesion, the surgeon chose a specific surgical approach according to the characteristics of the left main bifurcation lesion. The surgical approaches involved in this study included the following: (1) crush technique, (2) culotte technique, (3) T-stenting technique, and (4) V-stenting technique. After PCI, the platelet glycoprotein IIb/IIIa receptor antagonist (5–12.5 mg) was given according to the specific conditions of the patient. Besides, these patients received long-term statins while taking routine DAPT for at least 12 months.

In this study, DCB had a paclitaxel/iohexol matrix coating on the Bingo Drug-Coated Balloon (Bingo™, Yinyi Biotech, Dalian, China). To avoid geographic mismatch, the length of the DCB catheter was designed to exceed that of the target lesion by at least 2 mm. DCB diameters were adapted to reference vessel diameters with a balloon-to-vessel ratio of 0.8–1.0. The recommended inflation time was at least 30 seconds at >10 atm. New-generation DESs were implanted if DCB-only outcomes were unsatisfactory due to severe residual stenosis or dissections.

### 2.4. Measurement Indicators and Clinical Endpoints

Quantitative coronary angiography (QCA) software was used to analyze the CAG data of all patients, including the minimum lumen diameter (MLD) of each SB vessel at the left main bifurcation before PCI, immediately after PCI, and during CAG follow-up. Also, the corresponding degree of lumen stenosis and LLL at the target lesion were calculated separately. Lumen restenosis at the target lesion during follow-up refers to the degree of lumen stenosis of the vessel segment after PCI treatment ≥50% during CAG re-examination.

At least 6 months after PCI, the patients were suggested to return to the hospital for CAG re-examination. All patients were followed up for one year by reviewing their hospitalization medical records, outpatient return visits, and telephone follow-ups. The primary endpoints were LLL and lumen restenosis at each target lesion during follow-up angiography. The secondary endpoints were the major adverse cardiovascular events (MACE) that occurred during the patients' hospitalization and follow-up, including TLR, MI, and cardiac death. If there were multiple MACEs for the same patient, they were classified and recorded separately.

### 2.5. Statistical Analysis

SPSS V25.0 software was used for statistical analysis. Quantitative data with a normal distribution were expressed as the mean ± standard deviation (SD), and the Student *t*-test was used for analyzing the mean difference of two independent samples. Quantitative data with a non-normal distribution were presented as the median and interquartile range [*M* (*P*_25_, *P*_75_)], and the Wilcoxon rank-sum test was used for comparison between the two groups. Categorical data were expressed as a percentage (%), and the chi-square test was used for comparison between categorical values. For a 2 × 2 table, if the number 1 ≤ *T* < 5, the continuity corrected chi-square test was used; otherwise, the Fisher exact probability test was used. A two-sided test with a *P* < 0.05 was considered statistically significant.

## 3. Results

### 3.1. Baseline Patient Characteristics

A total of 100 patients were enrolled in this study, including 74 males (74%) and 26 females (26%), ranging from 30 to 85 (63.25 ± 10.29) years old. Among them, 71 cases (71%) and 29 cases (29%) were diagnosed as unstable angina pectoris and stable angina pectoris, respectively. Some patients had complications, including 44 cases (44%) of hypertension, 27 cases (27%) of diabetes, and 7 cases (7%) of hyperlipidemia. In the past medical history, 45 cases (45%) had a history of smoking, 8 cases (8%) had a history of MI, and 17 cases (17%) had a history of PCI. The LVEF was 62 (60–64%). Among the 100 patients, half (50%) received MB stenting plus SB DCB treatment, and the other half (50%) received dual stent treatment. There were no significant differences in the basic information of the two groups of patients. The baseline patient characteristics are shown in Table 1.

### 3.2. Patient PCI-Related Information

Regarding the types of predilated balloons used in MB, the DES + DCB group used more spinous balloons and cutting balloons, while the 2-DES group used more semicompliant balloons (*P* < 0.05). However, there were no significant differences in the types of predilated balloons used in SB between the two groups (*P* > 0.05). In the selection and use of stents/balloons, the maximum inner diameters of DES and DCB used in MB and SB of the 2-DES group were slightly but significantly larger than those used in the DES + DCB group (*P* < 0.05).

In terms of PCI, 45 patients (90%) in the DES + DCB group adopted the strategy of first MB stent implantation and subsequent SB DCB dilation, and the other 5 patients (10%) used the strategy of first SB DCB dilation and then MB stent implantation. In the 2-DES group, the crush technique was used in 31 cases (62%), followed by the culotte technique, T-stenting technique, and V-stenting in 9 cases (18%), 9 cases (18%), and 1 case (2%), respectively. In both groups, all patients underwent the final kissing balloon inflation (FKBI), and there was no significant difference in kissing inflation pressure between the two groups (*P* > 0.05). The PCI-related data of the two groups of patients are shown in Table 2.

### 3.3. Patient QCA Analysis

The QCA analysis results of all patients before and immediately after PCI are shown in Table 3. There were no significant differences in the reference vessel diameters, preoperative MLD, and preoperative luminal stenosis at the left main bifurcation between the two groups (*P* > 0.05). The CAG was re-examined immediately after the operation. The results showed that the DES + DCB group had a smaller immediate postoperative MLD at the LCX ostium and a greater degree of residual lumen stenosis compared with the 2-DES group (*P* < 0.05).

At least six months after PCI, 26 patients (52%) in the DES + DCB group and 23 (46%) patients in the 2-DES group returned to the hospital for CAG re-examination. There was no significant difference in CAG follow-up time between the two groups (*P* > 0.05). The follow-up results of the QCA analysis are shown in Table 4. Compared to the 2-DES group, the DES + DCB group had lower LLL of the left main stem (*P* < 0.05), higher MLD in the LCX ostium (*P* > 0.05), and lower luminal stenosis and LLL (*P* < 0.05). During the follow-up visits, there was no significant difference in the incidence of restenosis at the target lesion between the two groups (*P* > 0.05).

### 3.4. One-Year Clinical Follow-Up Results

The 1-year follow-up results of the two groups of patients showed that there was no significant difference in the occurrence of MACE between the two groups (*P* > 0.05) (Table 5).

## 4. Discussion

In recent years, with the sustained improvement of PCI technology, the interventional treatment of LMCA disease has made significant progress. A large number of clinical studies have proven that in certain types of patients, PCI is a reliable option for the treatment of left main disease [3, 4, 13, 14]. At present, the biggest difficulty in the treatment of left main disease with PCI is the treatment of left main bifurcation lesions. So far, the main interventional strategies for PCI treatment of left main bifurcation lesions include single-stent strategies and double-stent strategies. Each of them has advantages and disadvantages, and there is no single strategy available for all types of bifurcation lesions. DCB is a new treatment strategy for coronary artery disease, which has been generally recognized in the treatment of in-stent restenosis (ISR) and microvascular lesions [8, 15]. With the continuous accumulation of clinical practice, DCB has gradually been applied to coronary artery bifurcation lesions and macrovascular lesions and has shown reliable results. All of these provide a theoretical and practical basis for the hybrid DES (MB)-DCB (SB) approach shown in this study to treat the left main bifurcation lesions.

All patients in this study underwent re-examination of CAG immediately after PCI. The results showed that there was no significant difference in the immediate postoperative MB MLD of the two groups, but the MLD of the DES + DCB group at the SB ostium was smaller than that of the 2-DES group. In addition, the degree of residual lumen stenosis was higher in the DES + DCB group. This is because DCB, as an antiproliferative drug carrier, does not have the function of expanding and supporting the lumen. Therefore, the immediate effect of DCB on the treatment of SB ostium largely depends on whether the predilation is sufficient. In this study, to avoid artery dissection and unnecessary SB stent implantation, when using DCB in the SB, surgeons usually conservatively choose the diameter of the predilated balloon and DCB. Therefore, the expansion of the SB ostium is relatively limited. In the dual stent strategy, in order to better adhere to the vessel wall and cover the lesion, the stents that match the inner diameters of the vessel will be selected. In addition, due to the lack of metal stent support, elastic retraction of blood vessels will inevitably occur after the SB DCB is placed. Therefore, the SB MLD immediately after PCI in the DES + DCB group was relatively smaller when compared to the 2-DES group.

Follow-up CAG results showed that the MLD at the SB ostium of the 2-DES group was lower than that of the DES + DCB group, and the degree of residual lumen stenosis was higher than that of the DES + DCB group. The main reason for this was the higher LLL of the SB ostium in the double stent group. In contrast, most patients (18/26) in the DES + DCB group showed an increasing trend in the MLD of SB ostium during the follow-up period, which is in agreement with previous reports [16]. Although the specific mechanism of this phenomenon is not yet clear, it may be related to plaque redistribution and positive vascular remodeling [17]. Additionally, follow-up CAG results indicate that the LLL of the distal left main stem in the DES + DCB group was lower than that in the 2-DES group, which may be related to the DES + DCB group using more scoring balloons (spinous balloons and cutting balloons) when predilating the MB. Besides, such a result may also be related to the application of POT. Foin et al. [18] found that after MB stent implantation, the use of a short balloon with a large inner diameter to optimize the expansion of the proximal MB stent can improve the adhesion of the proximal MB stent and restore the original anatomical structure of the blood vessel to the greatest extent. Another retrospective study based on optical coherence tomography showed that poor stent adherence can cause local hemodynamic changes and delayed intimal healing, thereby increasing the risk of local restenosis [19, 20]. Based on this, European experts agree that POT can optimize the treatment effect of bifurcation lesions and improve the prognosis, so it should be performed as a routine procedure during bifurcation stenting [7]. However, Rigatelli et al. found that in terms of postoperative blood flow improvement, not all types of double stents can benefit from POT, especially DK-crush and culotte techniques, which cannot significantly improve the hemodynamic parameters after POT [21]. In the present study, most patients received these two types of dual stents, which can partly explain the difference in follow-up angiography of the distal left main stem between the two groups. Generally, from the angiographic follow-up results, MB stent implantation combined with SB DCB therapy seems to have more advantages than traditional double stent in reducing SB LLL. In addition, using more spinous or cutting balloons during pretreatment may be beneficial to improve LLL.

After 1 year of follow-up, no significant differences were observed in the occurrence of TLR, MI, and cardiac death between the two groups, indicating that as for long-term prognosis, the new surgical strategy is not inferior to the traditional double stent PCI. It is worth noting that a consensus has been reached that when using a stent combined with a DCB strategy to treat coronary artery bifurcation lesions, SB DCB dilation should be performed before the stent is placed in the MB [22]. The reason is to avoid the difficulty of DCB passing through the stent mesh as well as to reduce the damage of the surface coating and the loss of the delivered drug during the process of DCB passing through the stent mesh. However, consistent with Jim's report [10], in this study, most patients had undergone different surgical procedures, and during the 1-year follow-up, no significant adverse effects on the prognosis were observed. This may be related to adequate SB pretreatment. Our results demonstrated that when using a combination of stent and DCB to treat left main bifurcation lesions, MB stent implantation and subsequent SB DCB dilation are a reasonable surgical sequence.

In summary, the combination of stenting and DCB in the treatment of left main bifurcation lesions is safe and effective. Compared with the traditional double stent strategy, the combination strategy has certain advantages in reducing the LLL of the SB ostium. This study has accumulated more experience for the interventional treatment of left main disease, but it also has certain limitations. First of all, as a retrospective study, there was a certain bias in the inclusion of patients. Secondly, the intracavitary imaging examination represented by IVUS has a high spatial resolution and can accurately identify the size and nature of the lesion. Using intracavitary imaging to guide interventional treatment of left main disease can optimize PCI results and improve prognosis [23]. Therefore, Chinese diagnosis and treatment guidelines recommend intracavitary imaging for class IIa and B indications [24]. However, in clinical practice, many patients refused to accept intracavitary imaging due to economic reasons, resulting in a low proportion of patients using precise PCI, which might have a certain impact on the surgical results. Finally, this study had a relatively small number of cases, a short follow-up time, and a low percentage of patients who completed the CAG re-examination during the follow-up period, which may also have had a certain impact on the results of the study. Therefore, the conclusions of this study need to be confirmed in larger randomized controlled trials and with longer follow-up periods.

## 5. Conclusions

The combination of stenting and DCB in the treatment of left main bifurcation lesions is safe and effective, and compared with the traditional double stent strategy, the combination strategy has certain advantages in reducing the LLL of SB ostium.

## Figures and Tables

**Table 1:** Baseline patient characteristics.

Characteristics	2-DES group (*n* = 50)	DES + DCB group (*n* = 50)	*P* value
Male [case (%)]	36 (72.0)	38 (76.0)	0.648
Age (years, mean ± SD)	64.74 ± 9.20	61.76 ± 11.17	0.149
Diagnosis [case (%)]			
Unstable angina	38 (76.0)	33 (66.0)	0.271
Stable angina	12 (24.0)	17 (34.0)	
With hypertension [case (%)]	18 (36.0)	26 (52.0)	0.107
With diabetes [case (%)]	17 (34.0)	10 (20.0)	0.115
With hyperlipidemia [case (%)]	5 (10.0)	2 (4.0)	0.433^*∗*^
Smoking history [case (%)]	20 (40.0)	25 (50.0)	0.315
MI history [case (%)]	5 (10.0)	3 (6.0)	0.712^*∗*^
PCI history [case (%)]	7 (14.0)	10 (20.0)	0.424
LVEF [%, *M* (*P*_25,_*P*_75_)]	62.50 (58.75, 64.00)	62.00 (60.00, 64.00)	0.519

^*∗*^Comparing two data sets using the continuity corrected chi-square test. LVEF, left ventricular ejection fraction; MI, myocardial infarction; PCI, percutaneous coronary intervention.

**Table 2:** Procedural characteristics.

Characteristics	2-DES group (*n* = 50)	DES + DCB group (*n* = 50)	*P* value
Medina type [case (%)]			
1, 1, 1	41 (82.0)	44 (88.0)	0.774^#^
1, 0, 1	1 (2.0)	1 (2.0)	
0, 1, 1	8 (16.0)	5 (10.0)	
Preoperative SYNTAX score (*M* (*P*_25_, *P*_75_))	30.00 (26.75, 33.00)	28.00 (26.00, 30.00)	0.064
Multivessel disease [case (%)]			
Single vessel disease	0 (0.0)	0 (0.0)	0.806
Double vessel disease	11 (22.0)	10 (20.0)	
Triple vessel disease	39 (78.0)	40 (80.0)	
IABP [case (%)]	0 (0.0)	1 (2.0)	1.000
Temporary pacemaker [case (%)]	0 (0.0)	0 (0.0)	—
IVUS [case (%)]	14 (28.0)	14 (28.0)	1.000
OCT [case (%)]	0 (0.0)	0 (0.0)	—
FFR [case (%)]	0 (0.0)	0 (0.0)	—
MB predilated balloon type [case (%)]			
Semicompliant balloon	24 (48.0)	12 (24.0)	0.019
Spinous balloon	16 (32.0)	17 (34.0)	
Cutting balloon	10 (20.0)	21 (42.0)	
SB predilated balloon type [case (%)]			
Semicompliant balloon	36 (72.0)	34 (68.0)	0.139
Spinous balloon	11 (22.0)	7 (14.0)	
Cutting balloon	3 (6.0)	9 (18.0)	
Maximum inner diameter of MB (mm, *M* (*P*_25_, *P*_75_))	4.00 (3.50, 4.00)	3.50 (3.50, 4.00)	0.016
Maximum inner diameter of SB stent/balloon (mm, *M* (*P*_25_, *P*_75_))	3.00 (2.50, 3.50)	2.75 (2.50, 3.00)	0.007
DES + DCB [case (%)]			
SB DCB dilation first		5 (10.0)	
MB stent implantation first		45 (90.0)	
Two-stent PCI [cases (%)]			
Crush	31 (62.0)		
Culotte	9 (18.0)		
T-stenting	9 (18.0)		
V-stenting	1 (2.0)		
Inflation pressure (atm, *M* (*P*_25_, *P*_75_))	8.00 (8.00, 10.50)	8.00 (8.00, 8.50)	0.111

^#^Comparing two data sets using the Fisher's exact probability test. IABP, intra-aortic balloon pump counterpulsation; IVUS, intravascular ultrasound; OCT, optical coherence tomography; FFR, fractional flow reserve.

**Table 3:** Comparison of immediate postoperative effects between the 2-DES and DES + DCB groups.

Parameters	2-DES group (*n* = 50)	DES + DCB group (*n* = 50)	*P* value
Follow-up interval (month, *M* (*P*_25_, *P*_75_))	12.00 (10.00, 12.00)	11.00 (6.00, 12.00)	0.153
Left main stem			
Reference vessel diameter (mm, *M* (*P*_25_, *P*_75_))	4.03 (3.61, 4.17)	3.70 (3.56, 4.11)	0.123
Preoperative MLD [mm, *M* (*P*_25_, *P*_75_)]	1.35 (0.79, 1.78)	1.08 (0.74, 1.64)	0.238
Preoperative luminal stenosis [%, *M* (*P*_25_, *P*_75_)]	63.34 (55.82, 79.76)	70.99 (55.31, 81.10)	0.410
Immediate postoperative MLD [mm, *M* (*P*_25_, *P*_75_)]	3.63 (3.25, 3.75)	3.36 (3.17, 3.65)	0.125
Immediate postoperative luminal stenosis [%, *M* (*P*_25_, *P*_75_)]	10.53 (8.83, 11.60)	9.29 (6.71, 12.91)	0.348
Left anterior descending			
Reference vessel diameter (mm, mean ± SD)	3.36 ± 0.30	3.36 ± 0.33	0.942
Preoperative MLD [mm, *M* (*P*_25_, *P*_75_)]	0.87 (0.47, 1.10)	0.72 (0.54, 0.99)	0.543
Preoperative luminal stenosis [%, *M* (*P*_25_, *P*_75_)]	74.86 (66.76, 85.36)	78.57 (69.65, 84.14)	0.619
Immediate postoperative MLD (mm, mean ± SD)	3.06 ± 0.27	3.04 ± 0.32	0.744
Immediate postoperative luminal stenosis [%, *M* (*P*_25_, *P*_75_)]	8.21 (6.68, 9.50)	7.80 (6.08, 12.29)	0.853
Left circumflex			
Reference vessel diameter [mm, *M* (*P*_25_, *P*_75_)]	2.98 (2.55, 3.37)	2.78 (2.60, 3.02)	0.154
Preoperative MLD [mm, *M* (*P*_25_, *P*_75_)]	0.66 (0.44, 1.09)	0.73 (0.48, 0.98)	0.904
Preoperative luminal stenosis [%, *M* (*P*_25_, *P*_75_)]	76.70 (64.70, 86.20)	73.92 (65.94, 8 2.35)	0.610
Immediate postoperative MLD [mm, *M* (*P*_25_, *P*_75_)]	2.64 (2.22, 3.00)	2.20 (1.99, 2.47)	<0.001
Immediate postoperative luminal stenosis [%, *M* (*P*_25_, *P*_75_)]	11.11 (8.71, 14.33)	19.52 (14.61, 25.92)	<0.001

MLD, minimal luminal diameter.

**Table 4:** Comparison of CAG follow-up data.

Parameters	2-DES group (*n* = 23)	DES + DCB group (*n* = 26)	*P* value
Left main stem			
MLD (mm, *M* (*P*_25_, *P*_75_))	3.31 (2.88, 3.59)	3.23 (3.06, 3.37)	0.530
Luminal stenosis (%, *M* (*P*_25_, *P*_75_))	14.46 (13.27, 19.46)	13.66 (9.58, 19.73)	0.392
LLL [mm, *M* (*P*_25_, *P*_75_)]	0.17 (0.10, 0.29)	0.09 (0.03, 0.22)	0.037
Left anterior descending			
MLD [mm, *M* (*P*_25_, *P*_75_)]	2.88 (2.51, 3, 13)	2.88 (2.62, 3.08)	0.861
Luminal stenosis [%, *M* (*P*_25_, *P*_75_)]	12.57 (10.60, 17.77)	12.70 (9.83, 20.68)	0.812
LLL [mm, *M* (*P*_25_, *P*_75_)]	0.16 (0.09, 0.26)	0.16 (0.03, 0.34)	0.385
Left circumflex			
MLD [mm, *M* (*P*_25_, *P*_75_)]	1.80 (1.14, 2.54)	2.41 (2.02, 2.54)	0.031
Luminal stenosis [%, *M* (*P*_25_, *P*_75_)]	32.09 (18.85, 62.62)	16.71 (9.60, 22.47)	0.002
LLL [mm, *M* (*P*_25_, *P*_75_)]	0.43 (0.21, 1.59)	-0.17 (-0.31, 0.08)	<0.001
Restenosis [case (%)]			
Left main stem	1 (4.3)	2 (7.7)	1.000^*∗*^
Left anterior descending branch	1 (4.3)	2 (7.7)	1.000^*∗*^
Left circumflex	7 (30.4)	2 (7.7)	0.093^*∗*^

^*∗*^Comparing two data sets using the continuity corrected chi-square test. MLD, minimal luminal diameter; LLL, late lumen loss.

**Table 5:** Comparison of one-year MACE rates between the 2-DES and DES + DCB groups.

Parameters	2-DES group (*n* = 50)	DES + DCB group (*n* = 50)<	*P* value
TLR (case (%))	6(12.0)	3(6.0)	0.485^*∗*^
MI (case (%))	1(2.0)	0(0.0)	1.000^#^
Cardiac death (case (%))	0(0.0)	0(0.0)	—

^*∗*^The continuity corrected chi-square test; ^#^Fisher's exact probability test. TLR, target lesion revascularization; MI, myocardial infarction.

## Data Availability

The data used to support the findings of this study are included within the article.
